# Unlocking Happiness: Assessing the Monetary Value of Leisure Activities on Subjective Well-Being

**DOI:** 10.3390/healthcare11212884

**Published:** 2023-11-02

**Authors:** Yeongbae Choe, Jooa Baek

**Affiliations:** 1 Department of Tourism Management, Gachon University, Seongnam-si 13306, Republic of Korea; ychoe@gachon.ac.kr; 2 Department of Tourism Management, Jeju National University, Jeju-si 63243, Republic of Korea

**Keywords:** subjective happiness, leisure participation, quantification, well-being, compensating surplus, well-being valuation method

## Abstract

Improving the level of subjective well-being or happiness is often the primary goal for the government and its policy. Thus, knowing the value of leisure activities in improving the subjective well-being would hugely contribute to the policy-making process. This study explores the impact of participation in leisure activities on individual subjective happiness by applying the well-being valuation method to the national survey data collected in South Korea. Among the five leisure activities, participation in culture and arts, participation in sports, and tourism activities emerged as significant determinants of subjective happiness at the significant level of 0.05. At the individual level, participation in culture and arts exhibited the highest monetary value, while tourism activities have the highest monetary value at the national level, factoring in the total population and the participation rate across the country. These findings confirm the effect of leisure participation in improving subjective well-being and its corresponding monetary value and suggest that government should be appropriately utilizing the leisure policy to increase the frequency and type of leisure participation, thereby enhancing the level of happiness across the society. Results have significant implications for policy makers, practitioners, and academics in the domains of leisure and tourism.

## 1. Introduction

In general, leisure is defined as self-determined activities during free time [1] or activities that are not work-related or obligatory [2,3]. That being said, participating in leisure activities is entirely voluntary and enjoyable, undertaken for relaxation, entertainment, or self-fulfillment, and chosen freely [4,5,6,7,8,9]. Examples of leisure activities include hobbies, sports, socializing, and cultural events.

Participating in leisure activities, such as culture, leisure, sport, and tourism activities, has increasingly become a vital component of individuals’ lives, offering opportunities to relax, unwind, and pursue personal interests outside of work or other obligations [1,2,3]. Apart from the immediate enjoyment and pleasure that leisure activities provide, participating in leisure activities brings positive energy to one’s daily life and alleviates negative emotions. For example, those who may not experience success or recognition in other areas of their lives feel a sense of accomplishment and self-esteem [4,10,11,12,13]. In addition, immediate enjoyment and pleasure coming from leisure activities brings positive enduring emotions and a sense of meaning, thereby fostering positive attitudes toward life [1,8,14,15]. On the other hand, engaging in enjoyable and relaxing leisure activities helps deal with life’s challenges and helps restore a sense of balance and well-being by experiencing relief from stress and negative emotions [16,17]. Leisure activities facilitate social interaction and support that help foster a sense of belonging and connectedness [1,8,14]. Indeed, many leisure activities happen as a form of group-based activities, such as team sports, thereby fostering a shared identity and enhancing social cohesion.

Several attempts have been made to explain the effect of leisure activities and one’s subjective happiness and well-being. Carruthers and Hood [18] proposed the leisure and well-being model. In his model, the authors emphasized the importance of leisure experience and participation for one’s well-being, which is defined as the achievement of one’s full physical, cognitive, and social–emotional potential through successful, satisfying, and productive engagement. Similarly, a psychological model proposed by Newman, Tay, and Diener [10] explains a connection between leisure and one’s well-being. In their model, the structure of leisure including activities and the amount of time spent on leisure activities, as well as one’s subjective experience of leisure, such as the perceived frequency of in leisure activities and perceived participation, have an impact on individual well-being or subjective happiness. Both frameworks put forward the idea that participating in leisure activities triggers psychological processes, ultimately leading to an improvement in well-being and subjective happiness.

In recent years, the fields of psychology, sociology, leisure, and tourism have witnessed a significant surge in interest concerning the intricate relationship between leisure activities and one’s subjective well-being, particularly with respect to life satisfaction. This heightened curiosity is underpinned by an ever-expanding body of empirical research that delves into the multifaced aspects of this connection. For instance, a substantial body of research has consistently demonstrated the profoundly positive impacts of physical activities on both mental and physical health, ultimately contributing to enhanced well-being. Studies conducted by Bum, Yang, Choi [19], Hartman et al. [20], Kim and Choi [21], Sato, Inoue, Du, and Funk [22], and Van Cauwenberg et al. [23] have provided compelling evidence in support of the beneficial effects of physical engagement, including sport participation, physical exercise, and even the simple act of watching sports. Similarly, cultural activities have emerged as influential components that not only enhance psychological well-being, but also foster social cohesion. Well-documented investigations by Bakhshi, Fujiwara, Lawton, Mourato and Dolan [24], Fujiwara, Kudrna, and Dolan [25], and Lawton, Fujiwara, and Hotopp [26] underscore the pivotal role of cultural engagement. Activities such as attending music concerts, engaging in arts and crafts, and visiting museums have been linked to heightened psychological well-being and the strengthening of social bonds. In a related vein, tourism activities have been revealed to possess a distinctive capacity to alleviate depression, mitigate negative moods, and ultimately augment life satisfaction. The empirical work of Kang, Vogt, and Lee [27], McCabe and Johnson [28], and Vada, Prentice, Scott, and Hsiao [29] has solidified the understanding of the constructive impact of tourism on mental and emotional health.

While existing studies have confirmed both positive and negative relationships between leisure activities and subjective well-being [10,18,29], there remains an imperative to delve deeper into this complex relationship. The surging popularity of social tourism among academia, practitioners, and policymakers underscores the need for further investigation to establish the monetary value of such activities, especially when considering the substantial social costs involved. Nonetheless, earlier studies have not attempted to identify the types of leisure activities that are most beneficial for individual well-being, particularly in terms of monetary value. To address this gap in the literature, this study seeks to quantify the impact of participation in leisure activities on individual well-being and life satisfaction. Out research harnesses a nation-wide representative survey data collected in South Korea to gain a more nuanced understanding of the intricate relationship between a particular category of leisure activities and subjective well-being.

The overall goal of this study is to estimate the monetary impact that leisure participation has on subjective well-being, thereby understanding the value of engaging in leisure activities. To achieve this, priority should be given to the way of defining and measuring one’s subjective well-being. In tourism, subjective aspects of well-being are often connected to the specific psychological constructs depending on the study context, such as subjective well-being, happiness, life satisfaction, perceived quality of life, domain satisfaction, hedonic well-being, positive and negative affect [30,31]. Indeed, happiness and well-being are considered as conceptually overlapped [32] or even synonymous [31]. While previous studies have frequently used ‘subjective happiness’ and ‘life satisfaction’ interchangeably when assessing overall well-being, Fujiwara [33] argued that ‘happiness’ is a more appropriate term for this purpose based on its distinct conceptual definition. ‘Happiness’ predominantly captures an individual’s emotional state, affective experiences, and moods at a given moment, whereas ‘life satisfaction’ often incorporates an individual’s current emotions alongside their broader assessment of their overall life circumstances [33].

To rule out any possible confound effects, this study attempted to consider as many determinants of subjective happiness as possible, which is suggested by Fujiwara et al. [25]. First, demographic characteristics (i.e., gender, age, education level, marriage status, residence, and number of family members) were included as control variables. The literature on happiness and well-being in tourism and leisure fields repeatedly reported the effect of demographic characteristics on leisure participations and subjective happiness [27,33,34,35]. Second, subjective health status was considered; indeed, subjective health status is moderately associated with one’s happiness or life satisfaction [36,37,38]. Third, as leisure activities and subjective well-being is are associated, several leisure-related experiences and perceptions were considered in the model. For example, a voluntary work experience, leisure club participation, and recurring leisure experience participation were included. Earlier studies confirmed that a voluntary work experience has a strong effect on one’s happiness; therefore, the effect should be controlled for [27,39,40]. Furthermore, a regular-basis leisure activity participation (e.g., a leisure club, recurring leisure activity) should be differentiated with the effect of various leisure activity types on happiness [27,41]. Lastly, one’s perceived leisure participation (i.e., sufficiency of leisure expenses, sufficiency of leisure time—weekdays, and sufficiency of leisure time—weekend) was controlled in the regression model. These variables could be considered as a proxy for leisure constraint that have effects on happiness if they are insufficient [20,42,43].

We acknowledge that the choice of the existing dataset and study design may be influenced by the unique contextual factors, cultural considerations, and the specific situation in South Korea. However, we chose South Korea as a case study to fulfill the objectives of this research, recognizing its potential to serve as a foundation for future studies. The findings of our study are poised to offer substantial value to policymakers and practitioners in the leisure and tourism fields. This research can empower them with insights to better formulate social policies and design programs, effectively allocating financial resources to activities that offer the most significant benefits for individuals and society as a whole. In summary, this study contributes to filling a critical gap in the literature, striving to provide valuable guidance for the development of evidence-based policies and programs in the field of leisure and tourism.

## 2. Methods

### 2.1. Data Source

The data used in this study were obtained from the 2021 National Leisure Activities Survey (NLAS) of Korea, a publicly available dataset (https://shorturl.at/diqAZ (accessed on 30 March 2023)). This survey included nation-wide representative samples of Korean individuals; it consists of 10,049 participants over 15 years of age across the nation. The NLAS was originally designed and conducted by the Ministry of Culture, Sports and Tourism and the Korea Culture and Tourism Institute to investigate the annual trend that reflects changes in lifestyle, leisure activity (including culture, sports and tourism activities), and quality of life [42,44]. As the NLAS is one of the official statistics approved by Statistics Korea (Approval Number 113014), the NLAS used the stratified multi-stage cluster sampling method—that is, region, administrative district, and cluster, to ensure the representativeness of the respondents against the Korean population. The target time frame of leisure activities was between August 2020 and July 2021 and the survey period was September 2021 to November 2021 through one-to-one household visit interviews. After a series of reinvestigation and verification stages, the nonresponse adjustment was applied using the nonresponse adjustment coefficient and weighting procedure.

### 2.2. Sample Characteristics

Since the original NLAS data used the stratified multi-stage cluster sampling method using the region, administrative district, and cluster data and adjusted the weighting scheme using the ranking ratio method, the sample is well-represented for the entire Korean population. There were slightly more female respondents (50.52%) than male (49.48%). Slightly less than half of respondents were over 50 years (46.53%), while about similar proportion of respondents were aged between 20 and 40 years (47.63%). This indicates that the aging population in South Korea is increasing significantly in recent years, and preparations for the aging society should be made. Slightly more than half of respondents (56.74%) were married, followed by unmarried (29.45%), widowed or divorced (13.81%) groups. For the household income, KRW 3 million—KRW 4 million was the most frequently mentioned category (USD 2367—USD 3156, 18.87%), followed by KRW 4 million—KRW 5 million (USD 3156—USD 3945, 16.03%), and KRW 6 million and above (USD 4734, 17.22%). Slightly over three-quarters of respondents graduated with education higher than high school (high school: 39.08%, college and above: 38.22%). The residence of respondents is reported in Table 1, which is consistent with the population of each region in South Korea due to the data collection method.

### 2.3. Measures

This study aimed to quantify the effect of leisure activities on subjective happiness, thereby identifying the determinants of subjective happiness. To achieve this, the study utilized a set of different variables for taking WVA. For WVA, three types of variables were adopted from the 2021 NLAS. First, subjective happiness was measured as a single item. Participants were asked to indicate the level of happiness they feel on a scale of 1 to 10 (1 = very unhappy, 10 = very happy). While one’s subjective happiness and well-being are clearly separate concepts, this study considered subjective happiness being able to tap into the important aspect of participating in leisure activities. Subjective happiness indicates one’s affective state while well-being questions contain an evaluative judgment [25]. Possibly, culture and arts-, sports-, and tourism-related leisure activities could influence not only one’s eudemonic well-being but also one’s affective well-being and subjective happiness.

Second, participants were required to choose leisure activities they participated in over the past year. The survey questionnaire included a total of eight leisure activity groups consisting of eighty-eight detailed leisure activities, that is, culture and art viewing activities (e.g., art exhibition, museum, music concert), culture and art participation activities (e.g., photo taking, drawing, poem writing), sports viewing activities (e.g., watch sports game at the stadium, watch sports game via broadcasting), sports participation activities (e.g., basketball, golf, swimming), tourism activities (e.g., visit cultural relics, oversea trips, festival, camping), hobby and entertainment activities (e.g., collection, cooking, fishing), relaxation and rest (e.g., bath, nap, watch the TV), and social and other activities (e.g., volunteering, religious, social activities). However, in this study, three leisure types (i.e., hobby and entertainment activities, relax and rest, and social and other activities) were excluded due to significantly skewed responses and their impact on model identification. Most of respondents experienced such leisure activities (e.g., hobby and entertainment activities: 97.49%, relaxation and rest: 99.93%, social and other activities: 98.60%). Third, as suggested by Fujiwara et al. [25], equivalized household income was calculated using the following equation: $household\;income\times \sqrt{the\;household\;size\;}$. As household income was originally measured in categories, it was required to first transform the categorical variable to a continuous variable using the concept of the pareto curve [45].

For the application of the WVA, this study further considered several other demographic and individual characteristic variables. First, demographic characteristics (i.e., gender, age, education level, marriage status, residence, and number of family members) were included. Second, subjective health status was measured on a scale of 1 to 7 (1 = very unhealthy, 7 = very healthy). Third, as suggested by Fujiwara et al. [25], several leisure-related perception and experience variables were included. For example, respondents were asked to indicate whether they participated into a voluntary work experience [39,40], a leisure activity club [41] and any of recurring leisure activities [46] over the past year. All these variables were measured on a set of dichotomous-type (yes–no) questions. Moreover, respondents were additionally asked to indicate whether they perceived their leisure expenses, leisure time during the weekdays, and leisure time during the weekend as sufficient, measured on a scale of 1 to 7 (1 = very insufficient, 7 = very sufficient).

### 2.4. Well-Being Valuation Approach (WVA)

WVA is a technique that estimates the measures of welfare change (e.g., consumer surplus and equivalent surplus) based on survey data regarding participant experience as measured by the level of subjective well-being [24,25,33]. The foundation of WVA is known as the compensating variation approach, which is in the family of revealed preference [47]. WVA uses subjective measures to derive marginal substitution rates between a target non-market good/activity/service and household income [25]. Therefore, WVA is often utilized to estimate the monetary value of non-market goods and services that participants experienced, such as the benefits of leisure activity participation on subjective well-being in this study. WVA assigns a monetary value to targeted behavior or a specific circumstance [39]. This approach aims to quantify the monetary value of the benefits individuals receive from activities, enabling policymakers and decision makers to better understand the trade-offs involved in choices that impact well-being. Additionally, this method allows researchers quantification of the benefits that are mostly non-monetary in nature.

In WVA, subjective well-being is considered a useful proxy for an individual’s underlying utility and well-being, making a key assumption that welfare is observed through measures of subjective well-being (or happiness, life satisfaction) [24,25,33]. This assumption enables researchers to accurately estimate compensating and equivalent measures of value using the following function. Then, the value of non-market goods and services *Q* can be derived from the following equation:

SWB(Q, M),

where *SWB* is a direct SWB function in which *Q* = non-market goods and services and *M* = income.

$$SWB\left(Q^{0},M^{0}\right)=SWB\left(Q^{1},M^{1}-CS\right),$$

where superscripts 0 and 1 explain conditions before and after the provision of the non-market goods and services *Q*, *CS* denotes Consumer Surplus.

Measures of welfare change are derived from the marginal rate of substitution between the non-market goods and services and money (i.e., income) in the SWB function and are generally estimated using the coefficients from a regression model (regardless of its complexity). WVA offers several methodological and theoretical advantages compared to other valuation methods [24,48]. WVA helps researchers avoid problems such as protest values, strategic bias, hypothetical bias, and contextual influence (e.g., priming effects). Furthermore, the use of econometric analysis, such as multivariate regression or instrumental variable regression, helps reveal the monetary value of experiences, activities, or programs without directly asking survey participants.

Some applications of WVA have been developed in various fields. In culture studies, several attempts have been made to estimate the monetary value using WVA. Bakhshi et al. [24] measured the economic value of the national history museum in London, while Fujiwara [33] estimated the compensating surplus associated with visiting museums in one’s free time. Fujiwara et al. [25] also applied the same method to a UK national representative survey to estimate the effect of museums, libraries, and heritage sites visit on one’s life satisfaction, thus calculating the monetary value of such activities. Similar attempts can be found in sports and leisure studies. The monetary value of sport participation was estimated using an additional minute of sport per year by Downward and Dawson [49] and using the different frequencies of participation by Orlowski and Wicker [50]. Furthermore, the monetary value of participating in sports volunteering was captured by Thormann et al. [39] and by Lawton, Gramatki, Watt, and Fujiwara [40]. Furthermore, other topics, including public health and intervention [51], roadworks and flooding [52], and trust in government [48] have been applied in WVA for the purpose of estimating the effect of an certain event.

To sum up, the application of WVA enables researchers to evaluate the economic and social impacts of activities, policies, programs, or external conditions. The monetary value of nonmarket goods and services by WVA leads decision makers and policymakers with a more comprehensive understanding of social costs and benefits associated with policy options, thereby assisting them in making more informed decisions on ways to allocate resources and design programs that maximize the social benefits.

### 2.5. Model Specification

To apply the WVA, a multivariate regression analysis was used to investigate the relationship between one’s subjective happiness and leisure participations to estimate the monetary valuation of leisure activities. The use of a regression provided the extent to which leisure activities are associated with well-being impacts once other variables are controlled for. Then, the regression coefficients were used to quantify the effect of leisure activities on subjective happiness. The model specified below includes several determinants discussed in the literature review. The below equation describes the model specification for the current study:

$${Subjective\;Happiness}_{i}=\alpha +\beta_{1}{\;Income}_{i}+\beta_{2}{\;Leisure\;activities}_{i}+\beta_{3}{\;Control\;variables}_{i}+\varepsilon_{i},$$

where ${Subjective\;Happiness}_{i}$ is individual i’s subjective happiness over the past year; ${Leisure\;activities}_{i}$ is a list of leisure activities that the individual i undertook during the period; ${\;Control\;variable}_{i}$ is a vector of control variables and $\varepsilon_{i}$ is an error term.

After coefficients were derived, the impact of income ($\beta_{1}$) and the effect of leisure activities ($\beta_{2}$) were taken from the regression model, and these results were input in the below equation:

$$CS={Income}^{0}-e^{\left[\ln\left({Income}^{0}\right)-\frac{\beta_{2}}{\beta_{1}}\right]},$$

where *CS* is consumer surplus by participating in leisure activities; ${Income}^{0}\;$is an equivalized household income; $\beta_{2}$ is a regression coefficient of leisure activity and $\beta_{1}$ is a regression coefficient of household income.

## 3. Results

### 3.1. Study Variables

Table 2 describes leisure activity-related variables and control variables that are used in the analysis. The purpose of Table 2 is to show the descriptive statistics of variables used in the study. Respondents in our data indicated that they are moderately happy (7.01, out of 10). For the leisure activity participation, of total, about 46% of respondents participated in culture and art viewing activities, while only 15% were involved in culture and art participation activities. Indeed, actual participation in culture and arts would need additional effort, time, and resources compared to merely culture and art viewing activities. However, the ratio of sports viewers and sports participants in our sample was the same (50%). It is possible that sports participation includes a variety of activities ranging from basketball, golf, or swimming to jogging, yoga, Pilates, etc. Lastly, about three quarters (74%) of respondents participated in any form of tourism activities in the past year.

Several variables were included to derive unbiased effect of leisure activities as much as possible by controlling potential influencing factors. In total, only 4% of respondents and 6% of respondents participated in voluntary work experience over the past year and leisure activity club(s) over the past year, respectively. However, around one third of respondents (35%) had experience of recurring leisure activity participation over the past year. On average, respondents considered that their leisure expenses and leisure time, both on weekdays and on weekends are sufficient (4.37, 4.63, and 5.04, respectively). For subjective health status, respondents were more likely healthy (5.34 out of 7). Lastly, the number of family members was slightly below three.

### 3.2. Relationship between Leisure Activities and Subjective Happiness

Then, a multivariate regression analysis was performed to understand the relationship between leisure activities and subjective happiness. It should be noted that the results in Table 3 should not be directly interpreted as a causal relationship and the impact estimates of each leisure activity could be biased to some degree, depending on any potential confounding factors that are not included in the model [26]. Nevertheless, the fitted regression model was checked, and it was confirmed whether the model meets the assumption of linear regression.

Table 3 shows the results from our regression analysis with model specification presented earlier. Our fitted regression model explains 18.2% of the variance in individual’s subjective happiness (adjusted R2 = 0.182, df = 20, f = 112.54, *p* = 0.000). According to Fujiwara [33], a well-being regression for the WVA generally explained about 10% to 15% of the variance in the dependent variable, indicating that leisure participation and control variables used in our model indeed well explained one’s subjective happiness.

As expected, equivalent personal income (β = 0.22, *p* < 0.001) was a significant determinant of subjective happiness, meaning that higher income would increase one’s level of happiness. In addition, it was the strongest determinant among our variable of interest. Regarding the five leisure activity-related variables, culture and art participation activities (β = 0.11, *p* < 0.01), sports participation activities (β = 0.074, *p* < 0.05), and tourism activities (β = 0.07, *p* < 0.05) were statistically significantly associated with subjective happiness. Interestingly, two viewing activities (culture and arts, sports) were insignificant. Moreover, results from this analysis provide an interesting observation that leisure participation could enhance one’s happiness level, while leisure viewing activities could have no effect on one’s happiness level.

Most of control variables were statistically significant. Females were happier than males in our sample (β = 0.15, *p* < 0.001). Age had a U-shaped relationship with happiness in our data. A higher level of education also had a positively significant effect on happiness level (β = 0.12, *p* < 0.001). Married respondents (β = 0.08, *p* < 0.05) reported a higher level of happiness than others (either unmarried, widowed, or divorced). While residence was significant, this cannot be directly interpreted due to the measurement method. A set of dummy variables needs to be created for this variable (a total of 16 dummies); but to avoid the loss of degree of freedom, it was treated as a continuous variable in the current analysis. All three leisure-related experience variables (voluntary work experience: β = 0.23, *p* < 0.001; participation in a leisure activity club: β = 0.19, *p* < 0.001; experience of recurring leisure participation: β = 0.09, *p* < 0.001) were positively significant. Similarly, one’s perception about their leisure expenses and leisure time had a positive effect, but leisure time—weekend had no effect. This indicates that perception of sufficiency of leisure expenses and weekday leisure time increased happiness, while leisure time during weekend did not. Subjective health status had a strong effect on happiness level (β = 0.44, *p* < 0.001). Lastly, the number of family members was also a significant determinant of happiness level (β = 0.04, *p* < 0.001).

### 3.3. Quantifying the Participation in Leisure Activity

After obtaining coefficients from the regression analysis, the next step was to estimate monetary value, more specifically compensating surplus, for all leisure activity-related variables that were statistically significant in Table 3 using Equation (2). To achieve this, coefficients on each leisure activity and equivalent household income from the regression and the average of equivalent household income were applied to the equation. With this, monthly individual compensating surplus for leisure activities was estimated. It is worth noting that Powdthavee and van den Berg [53] indicated that the use of happiness may inflate the estimated value compared to the use of life satisfaction because one’s income generally has less effect on subjective happiness than on life satisfaction.

Engagement in culture and art participation activities had the highest value of KRW 971,253 (USD 757), followed by participation in sports activities (KRW 731,181, USD 570), and participation in tourism activities (KRW 697,654, USD 543). This study further considered the nation-wide participation rate to estimate total compensating surplus across the country (Table 4). While participation in tourism activities had the least monetary value at the individual level, it had the highest total compensating surplus due to the high participation rate (74.38%). Engagement in sports participation activities showed a higher total compensating surplus at the national level than culture and art participation, although participation in culture and art activities showed a higher compensating surplus at the individual level.

## 4. Conclusions and Implications

### 4.1. Conclusions

This study tried to address the gap in the literature by quantifying the effect of participating leisure activities on individual well-being and life satisfaction by applying the well-being valuation method. Specifically, this study utilized a nation-wide representative survey data collected in South Korea to better understand the relationship between a particular type of leisure activity and well-being and then estimated the monetary value of such leisure activities. The results indicate that leisure participation positively affects individual happiness level, with participation in culture and arts and sports activities having the highest value at the individual level, followed by participation in tourism activities. Although tourism activities were the least valued at the individual level, this variable had the highest total value at the national level, possibly due to its high participation rate across the country. These findings have important theoretical and managerial implications for policymakers, academics, and practitioners in promoting well-being through leisure activity participation.

### 4.2. Theoretical Implications

The findings of this study have theoretical implications. First, our analysis suggests that each type of leisure activity certainly has a differential impact on an individual’s well-being and happiness. This result may be due to differences in leisure constraints on participation for different activities [54,55,56]. Individuals may face various barriers and limitations when participating in leisure activities; then, their negotiation efficacy, leisure specialization, and other psychological factors (e.g., psychological continuum model or flow) may impact their level of happiness and well-being. Furthermore, individuals could feel happier to participate in a leisure activity than to simply join a viewing activity [57,58,59,60]. These findings contribute to the broader literature on leisure and well-being by highlighting the importance of considering a specific type of activity when examining the effects of activities on well-being.

Second, this study finds that various leisure-related factors, beyond just participation in in leisure activities, significantly influence subjective happiness levels, which is consistent with earlier studies [10,20,27,49]. Specifically, engagement in culture and art participation, sports participation, and tourism activity are found to be important determinants of individual subjective happiness. In our study, culture and arts and sport viewing activities are found to be non-influencing factors to subjective well-being. This result could be explained by the fact that such activities can be considered more enjoyable by the participants, but they do not make them happier. Alternatively, active participation indeed requires one’s time, efforts, and energy, thereby making people invested more than merely viewing the activities of others does. Additionally, almost all control variables (except for perceived sufficiency of leisure time on a weekend over the past year) are found to be significant determinants of subjective happiness, including joining a leisure club, participating in recurring leisure activities, or volunteering. These findings highlight the importance of considering various leisure-related factors when aiming to improve individual well-being.

Third, this study emphasizes the important role that leisure activities play in improving an individual’s well-being and happiness. Prior research has established the relationship between tourism and leisure behaviors and individual well-being and similar concepts (e.g., life satisfaction and happiness). However, this study reaffirms this connection using a monetary term through the WVA. By quantifying the value of leisure activities, the WVA demonstrates the usefulness of understanding the importance of a particular activity or behavior in monetary terms.

### 4.3. Practical Implications

The results from the current study provide some useful implications for practitioners and policymakers.

First, this study confirms the positive relationships between leisure participation and subjective well-being and similar concepts (e.g., happiness, life satisfaction), consistent with earlier studies [10,18,27,42,49]. The results suggest that the managers of leisure-related companies and policymakers should make efforts to increase the number of people who frequently participate in leisure activities, as it certainly helps well-being and happiness. For example, Seoul, South Korea recently launched a mobile-based self-health management app called ‘the wrist doctor 9988’ to encourage citizens living within the city to engage in leisure activities (e.g., walking, running) regularly. Such efforts can not only increase participation rates, but also significantly reduce unnecessary social welfare costs.

Second, government officials should consider building more leisure-related facilities within the city boundary to improve the perceived accessibility of these facilities. This would encourage citizens to participate in more leisure activities and ultimately increase the social welfare of the city [22,23]. Previous studies [61] have confirmed that having more leisure facilities enable citizens to participate in more leisure activities and feel more satisfied with their participation. The city may need to build more urban forests or park areas to provide opportunities for leisure activities [62,63,64]. In this regard, other built environments could also enhance an individual’s well-being and life satisfaction.

Third, this finding highlights the importance of social tourism. Social tourism has attracted significant attention from researchers and practitioners in the field of tourism and leisure, and policymakers have attempted to design tourism policies that use social tourism to improve social welfare across the country [28,29]. In South Korea, the travel voucher policy was introduced to provide domestic travel opportunities to selected recipients through vouchers, promoting their well-being and social welfare. Similarly, during the COVID-19 pandemic, Macau distributed coupons to its citizens to support small and medium businesses and alleviate ‘Corona Blue’, a form of pandemic-induced depression. Although the purposes of the two policies are slightly different, their goal is to enhance social welfare by promoting tourism and leisure activities. Based on the results of the current study, similar policies and debates should be further developed, providing more opportunities for people with limited resources to engage in leisure and tourism activities.

### 4.4. Limitations and Future Research Agenda

This study has some limitations that could be addressed in future research. First, the data used in this study were originally collected for other purposes, and, as a result, they contain limited variables that can be used for the current study. For example, the survey questionnaire only captures whether individuals participate in leisure activities but not the frequency, duration, or cost of these activities, or the strength of participants’ desire to participate. Second, similarly, the current study cannot verify the current results with other countries and cultures by comparison conducted between other datasets. Further study should be conducted with more leisure activities and comparing them with other countries and cultures to increase the generalizability of the results. Third, the current method and analysis did not incorporate any theories or key variables used in the field of leisure when estimating the value of leisure activities, which could affect the accuracy of the estimates. Future studies could consider incorporating such theories to estimate a more accurate value. Fourth, the sample in this study experienced a wide range of environmental conditions, such as geographical location and family composition (or number), which may have influenced their degree of participation in leisure activities and the impact on their well-being. Thus, future investigations should focus on the effect of external factors. Fifth, this study used multivariate regression analysis for the well-being assessment approach. However, more advanced techniques could reduce any potential bias in the estimates, providing a more accurate quantification. Lastly, the results of the current study should be only applicable to the specific context and population. Thus, interpretation should be cautious and follow-up studies should be needed to generalize to other population.

## Figures and Tables

**Table 1:** Demographic characteristics.

Characteristics	%	Characteristics	%
*Sex*		*Education*	
Male	49.48%	Elementary	11.33%
Female	50.52%	Middle School	11.36%
*Marriage status*		High School	39.08%
Unmarried	29.45%	College and above	38.22%
Married	56.74%	*Residence*	
Widowed or divorced	13.81%	Seoul	11.89%
*Age*		Busan	6.88%
15–19	5.85%	Daegu	5.77%
20 s	15.05%	Incheon	6.21%
30 s	14.85%	Gwangju	4.50%
40 s	17.73%	Daejeon	4.58%
50 s	18.91%	Ulsan	3.88%
60 s	14.70%	Sejong	2.00%
70 s and above	12.92%	Gyeonggi	13.10%
*Household income*		Gangwon	4.73%
Below KRW 1 million	9.94%	Chungbuk	4.78%
KRW 1 million–KRW 2 million	10.07%	Chungnam	5.59%
KRW 2 million–KRW 3 million	14.14%	Jeonbuk	5.09%
KRW 3 million–KRW 4 million	18.87%	Jeonnam	5.04%
KRW 4 million–KRW 5 million	16.03%	Gyeongbuk	6.20%
KRW 5 million–KRW 6 million	13.73%	Gyeongnam	6.78%
KRW 6 million and above	17.22%	Jeju	3.01%

**Table 2:** Study variables.

Study Variables	N	Min.	Max.	Mean	SD
Subjective happiness level	10,049	1	10	7.01	1.53
Culture and Art Viewing Activities	10,049	0	1	0.46	0.50
Culture and Art Participation Activities	10,049	0	1	0.15	0.35
Sports Viewing Activities	10,049	0	1	0.50	0.50
Sports Participation Activities	10,049	0	1	0.50	0.50
Tourism Activities	10,049	0	1	0.74	0.44
Voluntary work experience (over the past year)	10,049	0	1	0.04	0.20
Participation in a leisure activity club (over the past year)	10,049	0	1	0.06	0.24
Experience of recurring leisure participation (over the past year)	10,049	0	1	0.35	0.48
Perceived sufficiency of leisure expenses (over the past year)	10,049	1	7	4.37	1.26
Perceived sufficiency of leisure time—weekdays (over the past year)	10,049	1	7	4.63	1.39
Perceived sufficiency of leisure time—weekend (over the past year)	10,049	1	7	5.04	1.33
Subjective health status	10,049	1	7	5.34	1.20
Number of family members	10,049	1	7	2.70	1.25

**Table 3:** Determinants of subjective happiness.

Variables	Coefficients	t	Sig.
Constant	−0.73	−1.70	
*Variable of Interest*			
Equivalent Personal Income	0.22	8.45	***
Culture and Art Viewing Activities	0.05	1.60	
Culture and Art Participation Activities	0.11	2.65	**
Sports Viewing Activities	−0.01	−0.16	
Sports Participation Activities	0.07	2.40	*
Tourism Activities	0.07	2.10	*
*Control variables*			
Gender (1 = Female)	0.15	4.83	***
Age	−0.02	−3.70	***
Age^2^	0.00	5.03	***
Education	0.12	5.79	***
Marriage (1 = married)	0.08	2.36	*
Residence	0.01	3.69	***
Voluntary work experience (over the past year)	0.23	3.18	***
Participation in a leisure activity club (over the past year)	0.19	3.24	***
Experience of recurring leisure participation (over the past year)	0.09	2.98	***
Perceived sufficiency of leisure expenses (over the past year)	0.12	10.55	***
Perceived sufficiency of leisure time—weekdays (over the past year)	0.06	3.94	***
Perceived sufficiency of leisure time—weekend (over the past year)	0.02	1.04	
Subjective health status	0.44	31.78	***
Number of family members	0.04	3.67	***
Obs.	10,049		
Adj. R^2^	0.182		

*: *p* < 0.05, **: *p* < 0.01, ***: *p* < 0.001.

**Table 4:** Monetary value of leisure activities.

Type of Leisure Activities	Individual Compensating Surplus (Monthly)	Participation Rate (Nation-Wide)	Total Compensating Surplus (Nation-Wide)
Culture and Art Viewing Activities	- ^a^	-	-
Culture and Art Participation Activities	971,253	14.72%	6,507,102,795,169
Sports Viewing Activities	- ^a^	-	-
Sports Participation Activities	731,181	50.36%	16,759,385,313,166
Tourism Activities	697,654	74.38%	23,618,052,594,843

^a^ denotes ‘Not significant’.

## Data Availability

The raw data used in this study are publicly available via Micro Data Integrated Service (https://doi.org/10.23333/P.113014.001).
